# Milk Impurities

**Published:** 1891-11

**Authors:** 


### MILK IMPURITIES.

At the recent International Congress of Hygiene and Demography, held in England, Dr. Ostertag (Stuttgart) read a paper on the inspection of milk supplies with regard to the prevention of disease. He said that most countries had till now paid but slight attention to the sanitary question of the milk supply, and had contented themselves with forbidding the sale of adulterated milk or of milk from diseased animals, without taking proper steps to ensure obedience to their orders. A praiseworthy exception to this was to be found in the Italian law of provision supplies of August 3, 1890. It was the undeniable duty of the State to see that only pure milk entered the market. The consumer was not in a position to guard himself against the manifold dangers which attended the consumption of milk, which might contain the most harmful ingredients in spite of white color and sweet taste. Such milk could only be banished from the market if the milk supply was controlled by government officials. Only pure milk could be tolerated in the market—that was, milk obtained with the greatest cleanliness from healthy animals, and possessing normal physical qualities and a certain degree of strength. For sanitary reasons the following kinds of milk must be excluded from the market: 1. Milk which, without being necessarily prejudicial to health, was peculiar in color, taste or consistence—nauseous milk; 2. all milk that was prejudicial to health or which was suspected on good grounds of being so. To the first group belonged colostral milk, blue, red and yellow milk; further, slimy, thready, bitter, salt, as well as abnormally smelling milk, and milk that had been made impure by mud or other substances. The milk of animals that had been fed on poisonous fodder, or that had been treated with certain medicaments, and of those suffering from tuberculosis, malignant pustule, cow pox, aphthæ, or generally ill in consequence of some process inducing ulceration or ichor, must be regarded as prejudicial to health. The possibility of milk being of a hurtful nature was suggested in all the other feverish ailments common to milk-yielding animals, as also by the different forms of inflammation of the udder. Again, milk which had already been drawn might become infected by immediate contact with sick persons (typhus fever, cholera, &c.), or through being kept in rooms where such persons were. Finally, through being carried in unsuitable (metal) vessels, injurious substances might find their way into the milk. Professor Brown alluded to the sources of contamination of milk, and he mentioned that he had isolated organisms from perfectly healthy milk. In cases of compulsory closing of a dairy, compensation to the dairyman should be allowed. Dr. Armstrong (Newcastle) agreed as to the necessity of licensing dairy farms; he held that the milk of tuberculous cattle should be forbidden, and both tuberculous and parturient cows should be notifiable. Professor Wally said it was a great mistake for children to use the milk from one cow only. He agreed that tuberculosis should be scheduled.

